# Functional Molecular Plasma Biomarkers of Inflammation and Repair in Kidney Disease Progression in Gum Arabica Modality of CKD

**DOI:** 10.3390/ijms27020973

**Published:** 2026-01-19

**Authors:** Sameeha AlShelleh, Maysa Suyagh, Hussein Alhawari, Nailya Bulatova, Violet Kasabri, Ayman Wahbeh, Izzat Alawwa, Ashraf Oweis, Haneen Mustafa

**Affiliations:** 1 School of Medicine, The University of Jordan, Amman 11942, Jordan; 2 Jordan University Hospital, The University of Jordan, Amman 11942, Jordan; 3 School of Pharmacy, The University of Jordan, Amman 11942, Jordan; 4 School of Medicine, Jordan University of Science and Technology, Ramtha 22110, Jordan; 5 King Abdullah University Hospital, Jordan University of Science and Technology, Ramtha 22110, Jordan

**Keywords:** CKD, cystatin C, gum arabica, leucine-rich alpha 2 glycoprotein (LRG1), lipocalin 2, myeloperoxidase, orosomucoid 1 (alpha-1-acid glycoprotein 1 (ORM1)), plasminogen activator inhibitor 1 (PAI1), sirtuin 1, SOST–sclerostin 1, symmetric dimethylarginine (SDMA), uromodulin

## Abstract

Using colorimetric ELISA, this study aims to assess the impact of Gum Arabica (GA) consumption on functional molecular plasma biomarkers of chronic kidney disease (CKD) via a prospective cohort of GA-consumers (cases) vs. non-consumer (age- and CKD stage-matched) controls. Cohort’s hypertension (92.5%), dyslipidemia (64.8%), and diabetes mellitus (54.8%) were prevalent; the mean CKD duration was 6.94 years (SD 7.8) for both study groups. Comparable eGFR, sCr, ESR, CRP, HbA1c, FPG, UA, and fasting lipid parameters were in both study arms. In consumer cases, the mean duration of GA-consumption was 1.3 ± 1.1 (range 0.25–6) years with a mean dose of 1.7 ± 1.0 (range 0.5–6) spoons per day. Leucine-rich alpha 2-glycoprotein, plasminogen activator inhibitor 1, sirtuin 1, and SOST–sclerostin 1 were significantly (*p* value < 0.01) of lower concentrations, but lipocalin 2 and uromodulin were invariably (*p* value < 0.05) greater in the GA-consumer cases than those of controls. Strikingly, cystatin C, myeloperoxidase, orosomucoid 1, and symmetric dimethylarginine lacked any substantial variations in the GA-consumer cases vs. those in controls (*p* value > 0.05). Proportional correlations of CKD duration–PAI1 levels and sCr-lipocalin 2 levels but inverse correlations of orosomucoid 1-hypertension duration and SDMA-DBP were evident in cases.

## 1. Introduction

Chronic kidney disease (CKD) is estimated to impact 10–15% of the world’s population and is still on the rise. It is taken as a major public health concern characterized by a progressive decline in renal function and can occur in response to diabetes, hypertension, ischemia, or immune diseases. The demise of a critical number of nephrons can precipitate irreversible progression of CKD into end-stage renal disease (ESRD). Given the limited therapeutic options to prevent CKD progression, understanding the CKD molecular and mechanistic basis can be a pivotal challenge for the development of preventive and pharmacotherapeutic strategies [1]. In the initial phases, signs of chronic kidney disease (CKD), manifested as a significant decline in kidney function, often go unnoticed. If stages 1 to 3 are identified early, the advancement of CKD is modifiable, and hence, complications can be minimized. Stages 4 and 5, connected to considerable kidney damage typically leading to ESRD, can as such be avoidable.

The paramount literature on complementary and alternative medicine (CAM)-related self-management practices with chronic illnesses is available locally [2], regionally [3,4,5,6,7], and globally [8]. Ingestion of gum arabica (GA) especially altered lipid profiles, renal profiles, plaque, gingival scores, biochemical parameters, blood pressure, inflammatory markers, and adiposity. GA exhibited broadly anti-inflammatory, prebiotic, and antibacterial properties, successfully tackling sickle cell anemia, rheumatoid arthritis, metabolic disorders, periodontitis, gastrointestinal conditions, and kidney diseases [9,10,11,12,13,14,15,16]. Nevertheless, most of the studies conducted on GA were performed in rodents while a very limited number of studies were conducted on patients with a small sample size and short duration [9,10,11,12,13,14,15,16,17,18,19,20,21]. Principally, the effect of GA on decreasing serum creatinine and eGFR in CKD patients with diabetes was evident [20,21]; however, the sample size was small to prove causality. Other studies showed the effect of GA on decreasing oxidative stress and inflammation via decreasing levels of transforming growth factor beta 1 (TGFB1) [22] in CKD patients, thereby improving prognosis in patients with ischemic cardiac disease [23,24]. This sparked the idea to study the effect of this widely used supplement in our region on CKD pathogenesis and progression-related biomarkers [23,24,25,26,27]. Importantly, the value of using newer plasma and urine biomarkers for better diagnosis and management of CKD was signified [28,29,30,31,32], thereby paving the way for molecular functional biomarkers not used routinely in clinical practice or prospective clinical trials [33,34,35,36]. Furthermore, the translational evidence of intricate pathomechanisms involved in the development and progression of CKD necessitates the use of multiple markers rather than just one to accurately reflect the various changes taking place during this condition. Limited predictive accuracy was linked to diagnostic CKD’s blood urea and serum creatinine (sCr) levels. Emerging technology of urinary proteomics, metabolomics, and transcriptome can serve as a novel strategy to improve the diagnosis, prognosis, and treatment of diabetic CKD [25,26,27,28,29,30,31,32,33,34,35,36]. It remains essential to validate their effectiveness, sensitivity, and specificity, as well as to lower the costs associated with the analyses. We compared the most studied markers between two groups of CKD patients, one of which is consuming GA(vs. the non-consumer controls) that has a promising effect on some clinical parameters in CKD like eGFR, lipid profile uric acid levels, blood sugar, hemoglobin levels, body fat, BMI, and inflammatory markers.

Cystatin C serum levels are associated with BMI and insulin resistance in pre-diabetic patients [37]. Considerably, serum cystatin C is useful in detecting individuals with CKD having mild decrease in estimated glomerular filtration rate (eGFR) compared to serum creatinine. It has the clinical utility of estimation of GFR [38], urogenital malignancy [39,40], and cerebrovascular disease [41].

Leucine-rich α-2-glycoprotein 1 (LRG1) is a recently discovered plasma factor involved in angiogenesis that can forecast rapid declines in eGFR and the advancement of albuminuria in individuals with type 2 diabetes mellitus (T2D) [42], as well as indicate the likelihood of proliferative diabetic retinopathy in T2D patients [43].

Myeloperoxidase (MPO) is a hemeprotein stored and released from neutrophils and macrophages as a pro-inflammatory substance during the inflammatory process and oxidative stress conditions [44]. MPO is gaining an increased interest as it has been widely used as a biomarker for cardiovascular diseases including acute coronary syndrome [45]. In a cross-sectional case–control study investigating the association of MPO in overweight patients with insulin resistance, inflammation, and a first-degree relative with T2D, eighty-four overweight patients were enrolled in the study and divided into two groups according to the presence or absence of insulin resistance. The results showed a significant correlation between the MPO level and insulin resistance (IR) and inflammation in patients who had first-degree relatives with T2D, which increases the risk of developing T2D [46]. Strikingly, in obese children with vitamin D insufficiency, MPO levels, as a measure of oxidative stress, were substantially elevated [47].

Neutrophil gelatinase-associated lipocalin (NGAL), also known as lipocalin-2 (LCN-2), serves as a marker for both acute and chronic kidney injury [48]. Remarkably, it has been shown to reflect early changes in kidney damage even when urinary micro-albumin levels remain undetectable [49]. Given the interplay between LCN-2 and fibroblast growth factor 23 (FGF23), LCN-2 may serve as a connection linking the progressive deterioration of kidney function to increased bone production of FGF23 [50]. Notably, LCN-2 expression has been observed to govern mitochondrial dynamics and functionality, which is particularly relevant since mitochondrial dysfunction has been identified as a factor contributing to the progression of CKD. Inflammation, disrupted iron homeostasis, and altered metabolic activity frequently accompany CKD and are linked to elevated levels of LCN-2 produced by the kidneys and FGF23 secreted by the bones. Increased levels of LCN2 significantly enhance FGF23 production and are associated with cardiac injury in both patients and animal models of CKD. LCN-2 variants represent promising biomarkers for CMD and CKD in close connection to its role in promoting CKD vascular calcification by aggravating VSMC ferroptosis [51,52].

Orosomucoid 1 or acute phase protein α1-acid glycoprotein (AGP) is a serum protein known to be an “acute phase reactant” that has recently been shown to be needed for the maintenance of normal capillary permeability in skeletal muscle and mesentery. Invariably, adequate serum orosomucoid concentrations are an obligatory requirement for glomerular permselectivity [53]. Urinary orosomucoid is taken as a potential marker of inflammation in psoriasis [54,55,56]. Most remarkably, orosomucoid was reported to mitigate AKI and its progression to CKD through its anti-inflammatory action [57,58].

Plasminogen activator-inhibitor-1 (PAI-1) is present in most aggressive and progressive kidney diseases [59]. Sevelamer, a non-calcium-based phosphate binder, significantly decreased both endothelial expression of receptor for advanced glycation end product (RAGE) and endothelial dysfunction biomarkers, interleukin IL-6 and IL-8 (*p* value < 0.001), monocyte chemoattractant protein-1 (MCP-1; *p* value < 0.01), and PAI-1 and serum amyloid A (*p* value < 0.005), compared with the advanced glycation end products (AGEs)/uremic serum treatment alone [60].

Situin-1 plays a role in inflammation development through proteins that are deacetylated by SIRT1 [61]. In terms of mechanism, sirtuin 1 exerts its protective effects on the kidneys by regulating metabolic balance and autophagy, countering apoptosis and oxidative damage, and reducing inflammation by deacetylating histones and various transcription factors including p53, forkhead box group O, nuclear factor-κB, and hypoxia-inducible factor-1α, among others. Additionally, certain microRNAs have been associated with the progression of diabetic nephropathy (DN) as they target the mRNA of sirtuin 1.

SOST–sclerostin, a factor produced by osteocytes, inhibits the Wnt/β-catenin signaling pathway. It prevents the differentiation and function of osteoblasts. As a result, sclerostin serves as a powerful inhibitor of bone formation and mineralization [62]. Furthermore, targeting sclerostin presents a promising therapeutic approach for osteoporosis. Uremia is marked by a simultaneous occurrence of abnormal bone mineralization and accelerated cardiovascular calcification (chronic kidney disease-mineral and bone disorder, CKD-MBD), linking skeletal and cardiovascular health—this phenomenon is known as the bone-vascular calcification paradox.

Symmetric dimethylarginine (SDMA) is a catabolite amino acid generated by proteolysis in most cells at a constant rate. Primarily, it is subject to renal clearance, hence advocated as a measurement of kidney function. SDMA is involved in the early detection of chronic cardiovascular disease and renal impairment [63] and is therefore considered as a secondary prevention biomarker of CKD [64,65].

Uromodulin (UMOD) is a protein that is exclusively synthesized by the kidneys [66]. The polymers of urinary UMOD (uUMOD) play a protective role against kidney stones and urinary tract infections (UTIs). Remarkably, UMOD has various functions in regulating salt reabsorption, maintaining cation balance, and managing hypertension and acute kidney injury (AKI) [67,68,69]. Consequently, it is recognized as one of the selected novel biomarkers for diagnosing CKD and predicting its prognosis [70]. UMOD knockout mice show greater mortality rates during sepsis, which is linked to the upregulation of serum UMOD [67,68,69].

Our utmost aim was to investigate the potential impact of GA-CAM on the novel plasma biomarkers of renal damage in Jordanian CKD patients. Therefore, it was inevitable to explore the plasma levels of novel functional molecular biomarkers (namely, cystatin C, leucine-rich alpha 2-glycoprotein, lipocalin-2, myeloperoxidase, orosomucoid 1, plasminogen activator inhibitor 1, sirtuin 1, SOST–sclerostin 1, symmetric dimethylarginine, and uromodulin) as potential indicators for risk stratification and prognostication, refining diagnostic and preventive/therapeutic approaches, and describing some obstacles that still need to be overcome in CKD patients.

## 2. Results

### 2.1. Clinicodemographic Characteristics of the Study Sample (Table 1)

**Figure 1:**
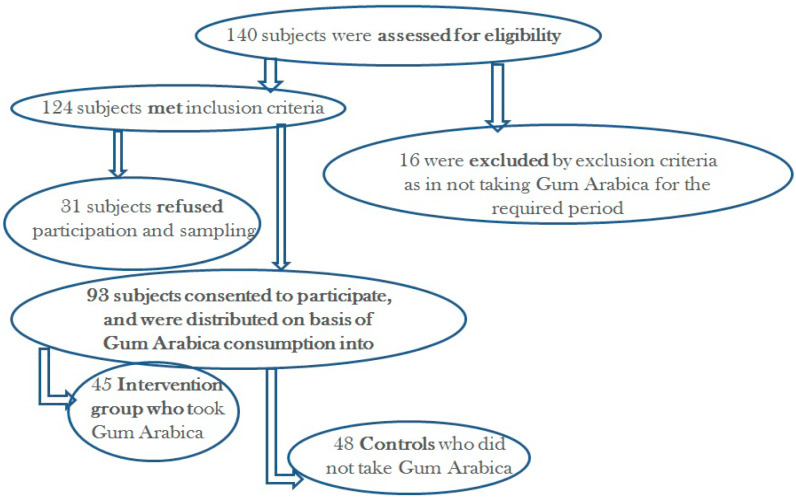
Recruitment flow chart.

In this cohort study, a total of 93 CKD patients, including 45 (48.4%) patients who received gum arabica (GA) (cases) in the past 6 months and 48 (51.6%) patients who did not receive GA(controls; age- and CKD stage-matched to the consumer cases) were enrolled (Figure 1). Demographic and clinical characteristics of the study participants with their comparison between the study groups are shown in Table 1. The mean age was 68.12 years. There were 56 males (60.2%) and 37 females (39.5%), with no significant differences in sex distribution between the study groups. Among chronic kidney disease (CKD) stages, the most prevalent were stage 4 (37.6%) patients, followed by stage 3a (20.4%) and stage 3b (19.4%), with no significant differences between the consumer cases and the controls (*p* value > 0.05). The mean CKD duration was 6.94 years, being similar between the two study groups (*p* value> 0.05). Among the concomitant medical conditions, the most prevalent was hypertension (92.5%), followed by dyslipidemia (64.8%), and diabetes mellitus (54.8%). The mean duration of GA use among the consumer cases of this study was 1.3 ± 1.1 (range 0.25–6) years and the mean dose was 1.7 ± 1.0 (range 0.5–6) spoons per day.

**Table 1:** Demographic parameters and medical history of study participants and their comparison between the groups (N = 93) at visit 1.

Characteristic		Total (N = 93)	GA Consumer Cases (N = 45)	Controls (N = 48)	*p*-Value
Age, years, mean (SD)		68.12 (10.01)	68.49 (10.52)	67.77 (9.60)	0.731 *
BMI, Kg/m^2^, mean (SD)		31.22 (5.78)	32.43 (6.50)	30.05 (4.76)	0.048 *
Sex, N (%) ^@^	Male	56 (60.2%)	26 (57.8%)	30 (62.2%)	0.676 **
	Female	37 (39.8%)	19 (42.2%)	18 (37.5%)	
CKD stage, N (%)	Stage 2	12 (12.9%)	6 (13.3%)	6 (12.5%)	0.912 ***
	Stage 3a	19 (20.4%)	9 (20.0%)	10 (20.8%)	
	Stage 3b	18 (19.4%)	7 (15.6%)	11 (22.9%)	
	Stage 4	35 (37.6%)	18 (40.0%)	17 (35.4%)	
	Stage 5	9 (9.7%)	5 (11.1%)	4 (8.3%)	
CKD duration, years, mean (SD)		6.94 (7.76)	6.49 (8.86)	7.37 (6.66)	0.590 *
Presence of diabetes mellitus, N (%)		51 (54.8%)	24 (53.3%)	27 (56.3%)	0.836 **
Diabetes mellitus duration, years, mean (SD)		17.14 (8.86)	16.77 (8.85)	17.48 (9.01)	0.777 *
Presence of diabetic neuropathy, N (%)		24 (25.8%)	12(26.7%)	12(25.0%)	1.00 **
Diabetic neuropathy duration, years, mean (SD)		5.84 (5.13)	7.13 (5.55)	5.20 (5.10)	0.533 *
Presence of hypertension, N (%)		86 (92.5%)	42 (93.3%)	44 (91.7%)	1.00 **
Hypertension duration, years, mean (SD)		13.49 (9.20)	13.69 (9.65)	13.30 (8.85)	0.847 *
Presence of dyslipidemia, N (%)		57 (64.8%)	29 (70.7%)	28 (59.6%)	0.371 **
Dyslipidemia duration, years, mean (SD)		8.71 (6.41)	9.00 (7.50)	8.44 (5.33)	0.773 *
Presence of coronary artery disease, N (%)		37 (39.8%)	16 (35.6%)	21 (43.8%)	0.526 **
Coronary artery disease duration, years, mean (SD)		6.08 (4.97)	5.08 (3.63)	6.67 (5.63)	0.340 *
Presence of heart failure, N (%)		7 (7.5%)	4 (8.9%)	3 (6.3%)	0.709 **
Heart failure duration, years, mean (SD)		8.33 (1.53)	8.00	8.50 (2.12)	0.879 *
Presence of thyroid/parathyroid disease, N (%)		14 (15.1%)	8 (17.8%)	6 (12.5%)	0.568 **
Thyroid/parathyroid disease duration, years, mean (SD)		5.74 (4.63)	6.67 (4.97)	4.81 (4.51)	0.512 *

* by independent-sample *t*-test; ** by Fisher’s exact test; *** by Chi-square test; @ within the group. Note. CKD: chronic kidney disease; SD: standard deviation.

### 2.2. Urinary Protein and Urine Glucose Tests Results at Visit 1 (Table 2)

Table 2 shows the results of urine tests for protein and glucose among the total sample and for the study groups at visit 1. More than a quarter of CKD patients (28.6%) had negative test results for urinary protein while relatively similar proportions (ranging between 14.3% and 22.0%) of patients had different degrees of proteinuria. Almost three quarters (74.7%) of CKD patients had negative results for urinary glucose, 14.3% had +1 urinary glucose, and a small proportion of patients (ranging between 3.3% and 4.4%) had higher degrees of glucosuria (from +2 to +4). There was no significant difference in distribution of proteinuria and glucosuria categories between the consumer cases and the controls (*p* values > 0.05 for all).

**Table 2:** Urinary protein and urine glucose results at visit 1.

Clinical Parameters		Total (N = 91), N (%)	GA Consumer Cases (N = 44), N (%)	Controls (N = 47), N (%)	*p* Value *
Total Protein (Dipstick Test)	Nil	26 (28.6%)	14 (31.8%)	12 (25.5%)	0.058
	+1	18 (19.8%)	6 (13.6%)	12 (25.5%)	
	+2	14 (15.4%)	3 (6.8%)	11 (23.4%)	
	+3	20 (22.0%)	13 (29.5%)	7 (14.9%)	
	+4	13 (14.3%)	8 (18.2%)	5 (10.6%)	
Urine Glucose (Dipstick Test)	Nil	68 (74.7%)	30 (68.2%)	38 (80.9%)	0.488
	+1	13 (14.3%)	9 (20.5%)	4 (8.5%	
	+2	3 (3.3%)	2 (4.5%)	1 (2.1%)	
	+3	3 (3.3%)	1 (2.3%)	2 (4.3%)	
	+4	4 (4.4%)	2 (4.5%)	2 (4.3%)	

* By Chi-square test. A “trace” reading on a dipstick test was approximately 100 mg/dL while higher notations like 1+, 2+, etc., indicate increasingly higher, approximate concentrations.

### 2.3. Comparison of Baseline Kidney Function and Clinical Biochemistry Tests Between Study Groups (Table 3)

At visit 1, there was no significant difference between the two study groups in estimated glomerular filtration rate (eGFR), with mean of 34.02 ± 17.72 for the consumer cases and 37.01 ± 16.76 for the controls (in mL/min/1.73 m^2^), with *p* value = 0.406, as assessed by the Chronic Kidney Disease Epidemiology Collaboration creatinine equation (eGFR/CKD-EPI). Evidently, comparable findings were obtained for both groups for ESR, CRP, HbA1c, and FPG.

**Table 3:** Comparison of EGFR, SBP, DBP, and SCr and their respective changes between the study groups at different assessment time points.

Clinical Parameter	GA Consumer Cases			Controls			*p* Value
	N	Mean	SD	N	Mean	SD	
eGFR/CKD-EPI (mL/min/1.73 m^2^)	45	34.02	17.72	48	37.01	16.76	0.406 *
ESR (mm/hr)	27	55.2	28.7	24	52.2	33.7	0.731 *
High sensitivity CRP (mg/dL)	25	20.4	36.0	27	22.9	28.4	0.787 *
HbA1c (%)	45	6.62	1.36	45	7.08	1.67	0.158 *
FPG (mg/dL)	45	129.6	64.5	47	140.1	73.1	0.470 *
sCr1 (mg/dL)	44	2.1691	0.9801	48	1.9306	0.9103	0.231 *
sCr2 (mg/dL)	45	2.2169	1.0718	48	2.0469	0.9052	0.412 *
ΔsCr2-sCr1 (mg/dL)	44	0.0223	0.3412	48	0.1163	0.2876	0.155 ^$^
sCr3 (mg/dL)	38	2.3037	1.3334	45	2.1636	1.0298	0.599 *
ΔsCr3-sCr2 (mg/dL)	38	0.887	0.3970	45	0.8000	0.3030	0.912 ^$^
ΔsCr3-sCr1 (mg/dL)	37	0.1157	0.5346	45	0.1958	0.2868	0.416 ^$^
SBP1 (mmHg)	42	145.52	16.793	39	137.64	19.45	0.055 *
SBP2 (mmHg)	44	144.55	15.992	45	136.11	19.49	0.028 *
ΔSBP2-SBP1 (mmHg)	42	−0.67	17.75	36	−1.67	3.03	0.807 ^$^
SBP3 (mmHg)	44	143.02	16.970	43	134.09	17.81	0.019 *
ΔSBP3-SBP2 (mmHg)	44	−1.52	19.42	43	−3.16	15.18	0.663 ^$^
ΔSBP3-SBP1 (mmHg)	42	−2.45	19.58	35	−4.31	17.25	0.658 ^$^
DBP1 (mmHg)	42	78.48	12.120	39	77.51	12.94	0.731 *
DBP2 (mmHg)	44	80.48	12.498	45	74.64	12.85	0.033 *
ΔDBP2-DBP1 (mmHg)	42	2.38	11.84	36	−2.83	9.30	0.036 ^$^
DBP3 (mmHg)	44	78.98	10.290	43	73.79	10.86	0.025 *
ΔDBP3-DBP2 (mmHg)	44	−1.50	12.29	43	−0.79	10.12	0.769 ^$^
ΔDBP3-DBP1 (mmHg)	42	0.76	12.18	35	−3.23	11.73	0.148 ^$^
UA1 (mg/dL)	41	7.3	1.5	46	9.2	15.6	0.447 *
UA2 (mg/dL)	41	6.8	1.6	38	6.6	1.4	0.592 *
ΔUA2-UA1 (mg/dL)	38	−0.5	1.7	36	−3.1	17.8	0.390 ^$^
HDL-C 1 (mg/dL)	40	41.7	13.3	42	40.3	9.2	0.570 *
HDL-C 2 (mg/dL)	30	42.2	12.2	31	43.1	12.5	0.770 *
ΔHDL-C 2-HDL-C 1 (mg/dL)	26	4.00	9.17	29	3.90	8.50	0.966 ^$^
LDL-C 1 (mg/dL)	40	93.4	33.8	42	106.6	37.4	0.098 *
LDL-C 2 (mg/dL)	30	103.1	32.2	31	109.0	46.0	0.569 *
ΔLDL-C 2-LDL-C 1 (mg/dL)	26	3.7	33.3	29	6.2	39.1	0.852 ^$^
TC1 (mg/dL)	40	150.8	38.3	42	168.0	44.4	0.065 *
TC2 (mg/dL)	30	165.3	36.3	31	166.0	52.4	0.954 *
ΔTC2-TC1 (mg/dL)	26	8.3	34.4	29	−2.0	47.4	0.359 ^$^
TG1 (mg/dL)	40	166.4	80.2	42	164.8	63.6	0.920 *
TG2 (mg/dL)	30	189.2	78.1	31	176.5	70.2	0.507 *
ΔTG2-TG1 (mg/dL)	26	−4.0	69.7	29	−3.5	57.5	0.979 ^$^

* by independent sample *t*-test; $ by paired-sample *t*-test; Δ: changes between respective visits. Note: sCr: serum creatinine; numbers 1, 2 and 3 indicate visits 1, 2, and 3, respectively. CRP: C-reactive protein; DBP: diastolic blood pressure; eGFR/CKD-EPI: Estimated Glomerular Filtration Rate, Chronic Kidney Disease Epidemiology Collaboration creatinine equation; ESR: erythrocyte sedimentation rate; FPG: fasting plasma glucose; HbA1C: glycated hemoglobin; HDL: high-density lipoprotein cholesterol; LDL: low-density lipoprotein cholesterol; SBP: systolic blood pressure; TC: total cholesterol; TG: triglycerides; UA: uric acid.

### 2.4. The Effect of Gum Arabica on Serum Creatinine, Clinical Chemistry Parameters, and Arterial Blood Pressure (Table 3)

**Figure 2:**
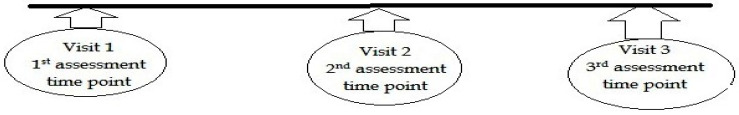
The study assessment timeline (with an average of 3 months apart assessment points) of three successive visits.

Serum creatinine (SCr) levels did not differ between the study groups at visit 1 and at the visits 2 and 3 (*p* values > 0.05 for all; Table 3). Furthermore, the changes in SCr between visit 2 and visit 1, visit 3 and visit 2, and visit 3 and visit 1 were comparable among the consumer cases as well as the controls equally (*p* values > 0.05 for all; Figure 2 and Table 3). Similar findings were essentially obtainable for serum levels of uric acid and fasting lipid profile parameters. Notably, SBP mean values (visits 2 and 3; respective *p* values of 0.028 and 0.018) and DBP mean values (visits 2 and 3; with respective *p* values of 0.033 and 0.025) for the consumer cases were reported to be significantly higher than those of controls.

### 2.5. The Effect of Gum Arabica on Blood Biomarkers of Kidney Damage (Table 4)

Table 4 demonstrates differences in blood biomarkers of kidney damage between the consumer cases and the controls at the end of the study. Obviously, LRG1 (*p* value = 0.006), PAI-1 (*p* value = 0.003), sirtuin 1 (*p* value < 0.001), and SOST–sclerostin 1 (*p* value < 0.001) were of significantly lower blood concentrations in the consumer cases than those of controls. Plasma concentrations of lipocalin-2 (*p* value = 0.018) and UMOD (*p* value < 0.001) were greater in consumer cases than those in controls. Consistently, in intergroup totality, cystatin C, MPO, orosomucoid 1, and SDMA lacked any significant variations in the plasma levels of the consumer cases vs. those of controls (*p* value > 0.05).

**Table 4:** Comparison of blood biomarkers of kidney damage between the study groups.

Parameters/CKD Plasma Molecular and Functional Biomarkers		GA Consumer Cases			Controls			*p*-Value *
		N	Mean	SD	N	Mean	SD	
1	Cystatin C (µg/mL)	45	55.30	6.95	43	55.08	4.91	0.868
2	LCN-2 (ng/mL)	45	427.82	211.66	43	314.16	231.70	0.018
3	LRG1 (µg/mL)	45	18.36	4.49	43	22.26	7.76	0.006
4	MPO (ng/mL)	45	2.77	0.70	43	2.73	0.67	0.757
5	Orosomucoid 1 (µg/mL)	45	67.53	28.19	43	55.96	26.37	0.050
6	PAI1 (ng/mL)	45	15.38	8.44	43	23.17	14.56	0.003
7	SDMA (µmol/L)	45	398.02	320.27	43	507.09	380.02	0.148
8	Sirtuin 1 (pg/mL)	45	116.73	49.84	43	212.58	60.16	<0.001
9	SOST–sclerostin (ng/mL)	45	9.02	1.40	43	11.52	3.46	<0.001
10	UMOD (ng/mL)	45	16.69	2.34	43	14.13	2.39	<0.001

* by independent sample *t*-test. Note: SD: standard deviation; LCN-2: lipocalin-2; LRG1: leucine-rich alpha 2 glycoprotein; MPO: myeloperoxidase; PAI1: plasminogen activator inhibitor 1; SDMA: symmetric dimethylarginine; UMOD: uromodulin.

### 2.6. The Effect of Gum Arabica on Correlations Between Blood Inflammatory and Cardiometabolic Biomarkers with Clinical Parameters (Table 5 and Table 6)

Notably, Table 5 and Table 6 illustrate that sirtuin substantially and inversely correlated (*p* values< 0.05) with the consumer cases’ HDL-C but proportionally with controls’ HDL-C. Exceptionally, orosomucoid negatively and pronouncedly related with the consumer cases’ HDL-C and likewise with both controls’ LDL-C and TC. Outstandingly, orosomucoid displayed a negative relation with thyroid-parathyroid dysfunctionality durations in GA naïve-controls of CKD modality. Furthermore, Leucine-rich alpha 2-glycoprotein (LRG1), SOST–sclerostin, and uromodulin associated directly and markedly with consumer cases’ LDL-C or consumer cases’ TG fasting levels (Table 5). Proportional myeloperoxidase–DBP linkage was significantly evident in the consumer cases. Moreover, symmetric dimethylarginine had significant disproportional correlations with controls’ DBP and fasting LDL-C (Table 6). Leucine-rich alpha 2-glycoprotein (LRG1) was of positive correlation with consumer cases’ HTN duration (Table 5). Similar outcomes were not evident among the rest of molecular plasma biomarkers of CKD (Table 5 and Table 6).

**Table 5:** Correlations between plasma biomarkers and clinical parameters in CKD GA consumer cases.

Parameters/CKD Plasma Functional Molecular Biomarkers	Sirtuin 1 (pg/mL)		Leucine-Rich Alpha 2-Glycoprotein (µg/mL)		SOST– Sclerostin (ng/mL)		Myeloperoxidase (ng/mL)		Uromodulin (ng/mL)		Orosomucoid 1 (µg/mL)	
	*r*	*p* Value	*r*	*p* Value	*r*	*p* Value	*p* Value	*r*	*p* Value	*r*	*p* Value	*r*
HTN Duration (years)			0.431 **	0.004								
DBP_1 (mmHg)							0.321 *	0.038				
HDL-C_1 (mg/dL)	−0.353 *	0.025										
HDL-C_2 (mg/dL)	−0.381 *	0.038									−0.378 *	0.039
LDL-C_1 (mg/dL)					0.316 *	0.047						
LDL-C_2 (mg/dL)			0.42 *	0.02								
TG_1 (mg/dL)									0.321 *	0.04		

*r*, correlation coefficient; * and ** statistically significant at *p* value ≤ 0.05 and ≤ 0.01.

**Table 6:** Correlations between plasma biomarkers and clinical parameters in CKD controls.

Parameters/CKD Plasma Molecular and Functional Biomarkers	Sirtuin 1 (pg/mL)		SDMA (µmol/L)		Orosomucoid 1 (µg/mL)	
	*r*	*p* Value	*r*	*p* Value	*r*	*p* Value
Thyroid/parathyroid disease duration (years)					−0.870 *	0.024
DBP_3 (mmHg)			−0.447 *	0.004		
HDL-C_2 (mg/dL)	0.439 *	0.019				
LDL-C_1 (mg/dL)			−0.330 *	0.040	−0.320 *	0.047
TC_1 (mg/dL)					−0.349 *	0.029

* Statistically significant at *p* value ≤ 0.05. Note: *r*: correlation coefficient; SDMA: symmetric dimethylarginine.

### 2.7. The Effect of Gum Arabica on Correlations Between Blood Inflammatory and Cardiometabolic Biomarkers with Kidney Function Parameters (Table 7 and Table 8)

Most notably in Table 7 and Table 8, in CKD cases consuming gum arabica, there were substantial and proportional associations of CKD duration with PAI1 levels (*r* = 0.424; *p* value = 0.049), as well as sCr with lipocalin 2 levels (*r* = 0.416; *p* value = 0.009).

**Table 7:** Correlations between plasma biomarkers and kidney function parameters in CKD GA consumer cases.

Parameters/CKD Plasma Molecular and Functional Biomarkers	PAI1 (ng/mL)		SOST–Sclerostin (ng/mL)		LCN-2 (ng/mL)		UMOD (ng/mL)		Orosomucoid 1 (µg/mL)		Cystatin C (µg/mL)	
	*r*	*p* Value	*r*	*p* Value	*r*	*p* Value	*r*	*p* Value	*r*	*p* Value	*r*	*p* Value
Duration (years)	0.424 *	0.049										
SCr_1 (mg/dL)							−0.341 *	0.024	0.313 *	0.039	−0.312 *	0.039
SCr_2 (mg/dL)									0.304 *	0.042		
SCr_3 (mg/dL)					0.416 **	0.009						
eGFR (mL/min/1.73 m^2^)									−0.320 *	0.032		
FPG (mg/dL)			−0.375 *	0.011								

* and ** statistically significant at *p* value ≤ 0.05 and ≤0.01.Note: *r*: correlation coefficient; LCN-2: lipocalin-2; PAI1: plasminogen activator inhibitor 1; UMOD: uromodulin.

**Table 8:** Correlations between plasma biomarkers and kidney function parameters in CKD controls.

Parameters/CKD Plasma Molecular Functional Biomarkers	Sirtuin 1 (pg/mL)		PAI1 (ng/mL)		LCN-2 (ng/mL)		UMOD (ng/mL)		SDMA (µmol/L)	
	*r*	*p* Value	*r*	*p* Value	*r*	*p* Value	*r*	*p* Value	*r*	*p* Value
BMI (Kg/m^2^)	0.363 *	0.018								
DM duration (years)			−0.515 *	0.008						
CKD duration (years)							0.449 **	0.003		
High sensitivity CRP (mg/dL)			−0.472 *	0.020						
HbA1C (%)									0.363 *	0.021
FPG (mg/dL)									0.379 *	0.013
Uric acid_2 (mg/dL)					0.453 **	0.008				

* and ** statistically significant at *p* values ≤ 0.05 and ≤0.01. Note: *r*: correlation coefficient; LCN-2: lipocalin-2; PAI1: plasminogen activator inhibitor 1; SDMA: symmetric dimethylarginine; UMOD: uromodulin.

Furthermore, leucine-rich alpha 2-glycoprotein positively correlated with the consumer cases’ hypertension duration (*r* = 0.431; *p* value = 0.004). Unequivocally in CKD controls, BMI positively correlated with sirtuin 1 level (*r* = 0.363; *p* value = 0.018) and diabetes duration associated pronouncedly and negatively with PAI1 (*r* = −0.515; *p* value = 0.08). On the other hand, orosomucoid 1 negatively correlated with hypertension duration (*r* = −0.870; *p* value = 0.024) while SDMA had significant disproportional correlations with DBP (*r* = −0.444; *p* value = 0.004). Similar outcomes were not evident among the rest of the molecular plasma biomarkers of CKD (Table 7 and Table 8).

## 3. Discussion

Our proposal aimed to characterize the molecular mechanisms by which this diverse battery of biomarkers can in totality assess the degree of chronic kidney disease (CKD) progression and the potential role of CAM in the delay of this progression. We expect this project to behighly likely able to lead to the discovery of new preventive/therapeutic strategies to target high-risk individuals with CKD for early treatment and preventive care via halting the onset and progression of CKD. With advancements in proteomics and metabolomics, new methods will enable the discovery of potential biomarkers for kidney diseases, facilitating early diagnosis of CKD and likely predictions of outcomes.

Our study included CKD patients at stages II–V who were not on dialysis. This was empowered by CKD clinical outcomes and with cross correlations with multiple molecular biomarkers of CKD progression [64,65,71,72,73,74,75,76]. Swaminathan et al. [77] detailed the challenging limitation of albuminuria and eGFR declines in the prediction of diabetic kidney disease (DKD) progression. Furthermore, the identification of crucial biomarkers based on the multifactorial pathogenesis of DKD can enable more targeted and effective diabetic treatment, as well as the prognostication of kidney disease progression and staging. This effectively expands the diagnostic opportunities to recognize patients at various stages of DKD progression, resulting in a decrease in the impact of DKD and ESRD [75,76]. The findings of this study, both individually and collectively, indicate that the combined application of multiple complementary biomarkers can enhance the precision in identifying kidney disorders and help define the molecular pathomechanisms of CKD, along with the corresponding interventions. This essentially presents a hugely invaluable targeting of high-risk individuals with CKD for clinicians and researchers for treatment and preventive care via halting the onset and progression of CKD [57,58,64,65,75,76,78,79]. Besides microRNAs, microvesicles, and exosomes as future CKD and DKD diagnostic tools [80], the implication of artificial intelligence (AI) in precision medicine can definitively facilitate the improvement of diagnosis/prognosis of microvascular complications [81]. The idea behind our study came from the fact that those patients in nephrology clinics, following advice from others in the community, are using GA, which is easily found and used by patients. A lot of them had the idea of consuming GA mainly for curing their CKD and less commonly to control blood pressure. The usual consumption dose is two tablespoons per day of GA powder dissolved in a cup of water and taken once in the morning and then in the evening. In our cohort, no effect was linked to eGFR, most likely due to other clinical parameters which affect the progression of CKD, as most of our patients had controlled blood sugar and blood pressure with minimal proteinuria, all of which can cause a rapid drop of eGFR. Also, the short follow-up time (three clinical visits over 6–9 months) was not enough to detect the change in serum creatinine eGFR and other lab parameters related to CKD complications or progression. To our knowledge, our study is the first to try to find a correlation of clinical and biomarker bases between the consumption of promising dietary supplement/possible future treatment like GA—which is turning into a hot topic nowadays—and CKD. We need to plan for future studies with a larger number of patients through different stages of CKD which allow more accurate results and a highly possible future treatment for CKD.

Homogenously, both consumer cases and controls had comparable levels of eGFR, serum creatinine, ESR, CRP, HbA1c, FPG uric acid, and fasting lipid profile parameters (*p* value > 0.05). Strikingly, sirtuin 1 (*p* value < 0.001), leucine-rich alpha 2-glycoprotein (*p* value = 0.006), plasminogen activator inhibitor 1 (*p* value = 0.003), and SOST–sclerostin 1 (*p* value < 0.001) were significantly and invariably of lower blood concentrations in GA consumer cases vs. those in non-GA controls. In the consumer cases of this study, 1.3 ± 1.1 (range 0.25–6) years was the mean duration of GA consumption with a mean dose of 1.7 ± 1.0 (range 0.5–6) spoons per day. These outcomes came as therapeutically associated with GA consumption. Moreover, several synthetic medications and natural substances have been discovered that enhance the expression and activity of sirtuin 1, providing protection against DN [82]. Recent findings revealed that the expression of sirtuin (SIRT1) declines in the proximal tubules prior to the onset of albuminuria in a mouse model of DN, where SIRT1 boosts the expression of the tight junction protein claudin-1 leading to albuminuria associated with DN [83,84].

Leucine-rich glycoprotein 1 is integral to the development of ocular neovascularization, cancer, diabetes, cardiovascular issues, neurological disorders, and inflammatory diseases [85]. Increased expression of LRG1 contributes to epithelial damage, acting as an enhancer of TGF-β signaling and driving tubulointerstitial fibrosis. Targeting LRG1 therapeutically could prove to be an effective approach to slowing the progression of kidney fibrosis in chronic kidney disease (CKD) [86]. It also serves a practical role as a new indicator of diastolic dysfunction [87]. Evidently, PAI-1 was reported to contribute to kidney scarring (fibrosis) and glomerulosclerosis [88,89]. In kidney pathology, PAI-1 regulates fibrinolysis and acts independently of proteolysis. Increased PAI-1 expression results in the accumulation of extracellular matrix (ECM) leading to DN, CKD, hemodialysis, peritoneal dialysis, and kidney transplantation [88,89]. Notably, in CKD patients, factors such as male sex, a history of cardiovascular disease (CVD), older age, higher phosphate levels, FGF23, PTH, lower eGFR, bicarbonate, calcitriol, and blood platelet counts were all significantly correlated with elevated serum sclerostin levels [90]. Consequently, it serves as a particularly relevant biomarker for dialysis patients suffering from CKD-MBD [91]. Additionally, the regulation of sclerostin is linked to various aspects of metabolic syndrome [91] as well as atherosclerotic lesions in patients with type 2 diabetes (T2D) [92]. Serum sclerostin concentrations are significantly greater in T2D patients compared to those with latent autoimmune diabetes in adults, regardless of metabolic syndrome or body mass index (BMI) [93]. Additionally, the calcifications observed within the aortic medial layer and renal vessels were significantly more pronounced when warfarin treatment was used in conjunction with anti-sclerostin antibody treatment [94].

Lipocalin-2 (*p* value = 0.018) and uromodulin (*p* value < 0.001) were substantially greater in consumer cases than those of controls. Of note, the clinical and translational significance of UMOD underscores its multifaceted role in renal ion transport and immune modulation, as well as its protective effects against UTIs and kidney stones; also, it can potentially be a systemic antioxidant [66]. Puthumana et al. [80] found that elevated levels of UMOD correlate positively with less significant declines in the estimated glomerular filtration rate (eGFR) and a reduced incidence of adverse kidney outcomes. Furthermore, UMOD is believed to be confined to the kidneys and the genitourinary system [67,68,69]. Additionally, UMOD displays immunomodulatory characteristics, with serum UMOD (sUMOD) being different from urinary UMOD (uUMOD), which attaches to harmful bacteria in the urine preventing infections and is also increased in kidneys undergoing repair following injury. Among CKD stages consistently prevalent in the consumer cases and controls (*p* value > 0.05), the study stage 4 (37.6% of total study population) was of predominant incidence, followed by stages 3a and 3b (20.4% and 19.4%, respectively). Invariably for both study groups (*p* value > 0.05), the mean CKD duration was 6.94 years (±SD 7.8) with approximately 75% of CKD patients of negative glucosuria while marginally 14.3–22.0% of them had proteinuria. Concomitantly, hypertension (92.5%), dyslipidemia (64.8%), and diabetes mellitus (54.8%) were consistently prevalent pathophysiologies in the study population. Remarkably, the mean values of SBP and DBP on the 2nd and 3rd visits (from respective baselines) for the consumer cases were reported significantly higher (*p* values < 0.05 in comparison to those of controls). LCN-2 is widely expressed across several tissues, such as the lung, heart, and kidney, and its expression is enhanced through the activation of the NF-κB, ERK, and JAK-STAT signaling pathways [48]. Rysz et al. [70] highlighted the potential usefulness of certain novel biomarkers in diagnosing chronic kidney disease (CKD) and predicting its outcomes. The combination of asymmetrical dimethyl arginine (ADMA), symmetric dimethylarginine (SDMA), uromodulin, kidney injury molecule-1 (KIM-1), and neutrophil gelatinase-associated lipocalin (NGAL) as well as miRNA, ncRNA, and lincRNA biomarkers can effectively reflect the various changes occurring throughout the progression of this disease. Most recently, LCN-2 has been recognized for its diagnostic capacity as an early indicator of renal failure in individuals with hypertension [95]. Conversely, a reduction in LCN-2 levels in mice with CKD leads to decreased FGF23 levels, improved cardiovascular outcomes, and extended lifespan [50]. Urinary variants of LCN-2, unlike those found in plasma or serum, have been linked to kidney function biomarkers ( *p* value < 0.05). Interestingly, the levels and ratios of LCN-2 variants in serum and plasma were notably associated with heightened risk of cardiometabolic disease (CMD), while those in urine had a significant correlation with renal dysfunction. Favorably, an incremental increase in uromodulin could be therapeutically associated with GA in CKD patients. Comparably, conclusive efficacies of lipocalin 2 were not applicable in the same cohort of CKD consumer cases of GA consumption. This can mostly be reflective of a mechanistically independent interplay of pathogenesis and downstream crosstalk in disease progression.

Consistently, myeloperoxidase, symmetric dimethylarginine (SDMA), orosomucoid 1, and cystatin C unjustifiably lacked any significant variations in the plasma levels of the consumer cases vs. those of controls (*p* value > 0.05). Despite 1.3 ± 1.1 (range 0.25–6) years as the mean duration of GA consumption with a mean dose of 1.7 ± 1.0 (range 0.5–6) spoons per day in the consumer cases, therapeutically, GA modulation of CKD pathomechanisms was not evidentially concluded in this prospective study. MPO activity and oxidized amino acids were taken for potential biomarkers in CKD and coronary artery disease [96]. Changes in the atherogenic index of plasma (AIP = Log10TG/HDL-C ratio) in children with end-stage renal disease (ESRD) are associated with the oxidative stress status and MPO concentration [97]. Additionally, MPO contributes to the progression of chronic inflammatory conditions such as atherosclerosis, neurodegenerative disorders, lung conditions, arthritis, cancer, and kidney ailments [98]. Furthermore, it serves as a connection between inflammation and oxidative stress in relation to cardiovascular disease (CVD) [99]. It is believed that CKD staging can be based on blood creatinine and SDMA concentrations in order to facilitate appropriate treatment and monitoring [74]. Remarkably, SDMA is enlisted among selected novel biomarkers in the diagnosis of CKD and the prediction of its outcome [70]. Urinary orosomucoid, or α1-acid glycoprotein, was found to be associated with the progressive CKD stage in patients with sickle cell anemia [54,55,56]. Outstandingly, increased urinary orosomucoid excretion (UOE) early in pregnancy predicted preeclampsia in women with pregestational type 1 diabetes independently of BMI, serum creatinine, smoking, and microalbuminuria. Reportedly, it is involved in the development of chronic allograft rejection after kidney transplantation [100]. Both serum creatinine and cystatin C levels increase with a decrease in eGFR. Impressively, serum cystatin C may be used to screen patients with poorly controlled diabetes mellitus or hypertension when the serum creatinine level is inconclusive/elusive [101].

The shortcomings of conventional renal dysfunction biomarkers such as serum creatinine have been extensively demonstrated in the literature. Thus, enhancing clinical evaluations with newer biomarkers like serum cystatin C could potentially improve disease monitoring and patient management [37,38,39,40,101]. It is mostly indispensable to fully understand how LRG1 contributes to the pathogenesis and pathophysiology of kidney diseases, both as a therapeutic target and as a molecular diagnostic indicator of GFR [42,43,85,86,87]. Targeted therapies focusing on LRG1 are expected to be employed in clinical trials and eventually integrated into clinical practice.

In a close cross-correlation with the clinical deterioration of GFR, LCN-2 was found to be directly associated with sCreatinine. Lipocalin 2 is produced by the kidneys in response to injury and has increased levels in CKD [48,49,50,51,52]. It serves as a biomarker for kidney damage and disease progression but contributes negatively to the condition by causing mitochondrial dysfunction, promoting inflammation, and elevating FGF23 levels [48,49,50,51,52,95]. LRG1 levels were found to be increased in urine, serum, or renal tissues of patients or experimental models with various kidney diseases, including DN [42,43,85,86,87]. Unlike UMOD, even with noteworthy therapeutic improvements in circulatory levels for GA consumer cases, therapeutically and substantially ameliorated LRG1, SOST–sclerostin, sirtuin 1, SDMA, and PAI1 lacked any significant associations with either sCreatinine or uric acid in our GA consumer cases cohort. Importantly, both orosomucoid 1 and lipocalin 2 had substantial correlations with sCreatinine. Furthermore, orosomucoid 1 was inversely and markedly related to GFR, while lipocalin 2 had a proportional and significant correlation with uric acid in non-supplemented GA-CKD controls and a marked correlation with sCreatinine in GA consumer cases. Basically, serum Orosomucoid 1 was not shown to be therapeutically modulated by GA (*p* value = 0.05 *), despite expectations of a pronounced decrease. Similarly, LCN-2 did not exhibit GA-effected therapeutically regulated effects and instead showed an increase.

Reduced levels of urinary uromodulin are linked to an increased likelihood of CKD and a rapid deterioration in kidney function, whereas elevated levels are indicative of improved kidney health [66,67,68,69]. In an ideal therapeutic outcome related to GA, a progressive and significant accumulation of uromodulin was associated with GA consumption in patients. Notably, uromodulin had a substantially disproportionate correlation with sCreatinine. Moreover, elevated levels of cystatin C in the bloodstream can suggest impaired kidney function and the existence of CKD [37,38,39,40]. It serves as a more precise or alternative marker to sCreatinine for estimating the eGFR, as it is not influenced by low muscle mass, making it useful for confirming a CKD diagnosis or more accurately stratifying patients by risk. UMOD shows an inverse relationship with sCreatinine, aligning perfectly with molecular diagnostics for CKD. Progressive decline in kidney function seen in advanced CKD can also result in reduced circulating myeloperoxidase levels due to the inhibitory effects of uremic toxins (as in p-cresol) on the enzyme [66,67,68,69]. Contrary to the expected therapeutic reduction in blood levels of both MPO and cystatin C, neither showed significant changes in expression levels in consumer cases with CKD-linked compromised GFR. Interestingly, cystatin C exhibited an inverse relationship with sCreatinine. Patients with CKD are often found to have elevated levels of sclerostin, which tend to decrease during the process of dialysis [62,90,91,92,93,94]. Elevated levels of SIRT1 are associated with a favorable prognosis in terms of renal protection [62,83,84]. Consequently, the activation of SIRT1 could be a viable therapeutic approach for CKD, as it may slow down the progression of the disease and its associated complications. Additionally, the blood test SDMA is utilized for the early diagnosis of CKD, as it rises with just a 25% reduction in kidney function, unlike sCreatinine, which typically does not show an increase until 75% of function is lost [63,64,65,74]. SDMA is considered a dependable measure of kidney function that is less influenced by muscle mass and has become a standard part of kidney health assessments to facilitate the early detection and staging of CKD. Plasminogen activator inhibitor-1 (PAI-1) plays a significant role in the CKD advancement [44,45,46,96,97,98,99]. While healthy kidneys contain low levels of PAI-1, its expression is notably elevated in diseased kidneys, promoting kidney fibrosis and damage [30]. In a group of GA consumer cases, PAI-1 levels positively correlated with the duration of CKD, but not sCreatinine. A similar correlation was found for UMOD in control subjects, aligning with the decline in GFR over the disease’s progression trajectory. The presence of orosomucoid in urine is associated with kidney damage and inflammation [54,55,56,100]. It is an acute phase protein produced by the liver, and higher levels in urine can signal early kidney injury that standard tests like proteinuria might miss. Using urinary orosomucoid might aid in the early detection and ongoing assessment of CKD. While significant declines in circulating levels were largely expected, increasing plasma concentrations of lipocalin 2 and orosomucoid (approaching statistical significance with *p* value = 0.05) were observed in GA consumer cases.

## 4. Materials and Methods

The Institutional Review Board (IRB) approval for conducting this prospective cohort study at the Jordan University Hospital (JUH) (169/2019)/nephrology outpatient clinics was obtained. Then, all patients fulfilling the inclusion criteria were recruited after explaining the purpose and the nature of the study and signing an informed consent form. Recruiting took place between October 2019 and October 2020. Blood samples (in lithium heparin used for single determinations) were obtained from CKD patients. *Gum Arabica* (*GA*), as a very highly standard refined product (raw plant material), was procured from a local herbalist.

Inclusion criteria were (a) adults 18–90 years old, (b) stable CKD stages II–V at the baseline visit, and (c) attending a nephrology clinic regularly for follow up.

Exclusion criteria were (a) pregnant ladies, (b) patients on renal replacement therapy, and (c) patients treated by complementary and alternative medicine (CAM) other than gum arabica.

Urinalysis for proteinuria and glucosuria was conducted routinely at clinics, and clinical chemistry analysis was frequented regularly for two or three successive visits for serum creatinine (sCr), glycated hemoglobin (HbA1c) and fasting plasma glucose (FPG), uric acid, erythrocyte sedimentation rate (ESR), and C-reactive protein (CRP), as well as fasting lipid profile parameters. Plasma levels of CKD molecular functional biomarkers were measured by enzyme-linked immunosorbent assay (ELISA) kits implementing respective manufacturers protocols with intra- and inter-assay accuracy and precision CV% 10–12% (human sirtuin 1, LRG1, ORM1, SDMA, and UMOD (MyBioSource, San Diego, CA, USA)), and cystatin C, lipocalin 2, MPO, PAI1, and SOST–sclerostin (Abcam, Cambridge, MA, USA).

Statistical analysis was performed using IBM SPSS© statistics version 22 (SPSS, Inc., Chicago, IL, USA). The independent sample *t* test, paired *t*-test, Chi-square, and Fisher’s exact analyses were performed as appropriate where *p* values < 0.05 were considered significant. Spearman or Pearson ranking of potential cross-correlations were assessed for the clinical and demographic parameters with investigated set of kidney function biomarkers.

## 5. Limitations

Small cohort of patients from one center and the limited number of biomarkers are initially major limitations. In addition, the ELISA for the one-time point of determination of plasma levels of markers is yet to be validated in a small cohort for its respective urinary and salivary levels and potential cross-correlations with eGFR [1,101] or albuminuria [102,103,104]. Limited resources could hinder the purchase of more pricy ELISA kits for matching assessments of both urine and saliva concentrations of all ten plasma functional molecular biomarkers for our small cohort. This study might have lacked control of the confounding factors of medication and diet, and the possibility for additional statistical methods in connection to its current non-interventional experimental design.

## 6. Concluding Remarks and Future Directives

As we highlight the importance of a collaborative/complementary approach to standardize the integrative use of promising new biomarkers, in CKD consumer cases taking GA, substantial and proportional associations of disease duration and PAI 1 levels, as well as sCr and lipocalin 2 levels, were found. In controls, the duration of CKD directly correlated with their uromodulin concentration.

Succinctly, we highlighted the shortcomings of traditional indicators of kidney injury as reflections of the underlying disease process taking place within the organ and its functional components, along with the exploration of anticipated trends in clinical and biochemical progression in patients with chronic kidney disease (CKD). Our results with positive biomarkers in favor of patients using GA will hopefully stimulate others to carry out similar testing and achieve favorable results that will benefit the field of evaluation and management of CKD. In reference to this frame, clinical utility of urinary adiponectin as a new diagnostic index for CKD could serve as a non-invasive urine test reducing patient burden. Of closely related significance, pro-inflammatory tumor necrosis factor-α (TNF-α) and IL-1, IL-6, and IL-18 were reported in strong relation with DKD [105,106]. Furthermore, in T2D patients, serum tumor necrosis factor receptors (TNFRs) were attributed with a much more marked association than urine TNFRs with both albuminuria and eGFR [101,102,103,104,105,106,107]. In addition, the development of a simple panel test may facilitate the usage of multiple biomarkers as a routine test to be frequented regularly in clinical settings of translational relevance, significance, and evidence. Taken together, it was evidently and consistently reported that a multimarker score could increase prognostic accuracy and reclassification of AKI and CKD in comparison to traditional clinical variables alone [80]. Indicators of injury to renal glomerular and tubular endothelial cells, like neutrophil gelatinase-associated lipocalin, can be incorporated into larger interventional trials. The use of random urine samples might raise the variability among individuals for each biomarker, and the findings may require verification through a 24 hr urine collection or first morning void urine samples. The use of multiple biomarkers can facilitate the development of new preventive/treatment strategies and risk stratification. Furthermore, the applicability of multiple biomarkers is valuable in fatality prediction in heart failure, diabetes mellitus, sickle cell anemia [1,75,76], and atrial fibrillation. Taken together, more data from larger randomized control studies are required to validate our findings.

## Data Availability

The raw data supporting the conclusions of this article will be made available by the authors on request.

## Abbreviations

AIP	atherogenicity index of plasma
AKI	acute kidney injury
BMI	body mass index
CAM	complementary and alternative medicine
CMD	cardiometabolic disease
CKD	chronic kidney disease
CRP	C-reactive protein
CVD	cardiovascular disease
DBP	diastolic blood pressure
DN	diabetic nephropathy
eGFR	estimated glomerular filtration rate
ELISA	enzyme-linked immunosorbent assay
ESR	erythrocyte sedimentation rate
ESRD	end-stage renal disease
FGF	fibroblast growth factor
FPG	fasting plasma glucose
GA	gum arabica
HbA1c	glycated hemoglobin
IL	interleukin
KIM-1	kidney injury molecule-1
LCN-2	lipocalin-2
LRG1	leucine-rich alpha 2 glycoprotein
MCP-1	monocyte chemoattractant protein-1
MPO	myeloperoxidase
NGAL	neutrophil gelatinase-associated lipocalin
OGTT	oral glucose tolerance test
ORM1	orosomucoid 1 (alpha-1-acid glycoprotein 1)
PAI1	plasminogen activator inhibitor 1
SBP	systolic blood pressure
sCr	serum creatinine
SD	standard deviation
SDMA	symmetric dimethyl arginine
sUMOD	serum uromodulin
TGFB1	transforming growth factor beta 1
T2D	type 2 diabetes
UACR	urine albumin-to-creatinine ratio
UMOD	uromodulin
uUMOD	urinary uromodulin
UTI	urinary tract infection
